# Histamine and TH2 cytokines regulate the biosynthesis of cysteinyl-leukotrienes and expression of their receptors in human mast cells

**DOI:** 10.1007/s00011-024-01974-6

**Published:** 2025-01-31

**Authors:** Patricia Gehlhaar, Katrin Schaper-Gerhardt, Ralf Gutzmer, Franziska Hasler, Till A Röhn, Thomas Werfel, Susanne Mommert

**Affiliations:** 1 Department of Dermatology and Allergy, Hannover Medical School, Carl-Neuberg-Strasse 1, 30625 Hannover, Germany; 2 Department of Dermatology, Johannes Wesling Medical Center, Ruhr University Bochum, Minden, Germany; 3 Immunology Disease Area, Novartis BioMedical Research, Novartis Pharma AG, Basel, Switzerland

**Keywords:** Atopic dermatitis, Mast cells, Histamine, TH2 cytokines, Biosynthesis of cysteinyl leukotrienes

## Abstract

**Introduction:**

In skin lesions of atopic dermatitis (AD), a chronic inflammatory skin disease, mast cells beyond other immune cells are present in increasing numbers. Upon activation, mast cells release a plethora of mediators, in particular histamine and leukotrienes, as well as chemokines and cytokines, which modulate the immune response of cells in their microenvironment and may influence mast cells in an autocrine loop. This study investigated the effects of histamine and TH2 cytokines on the biosynthesis of cysteinyl leukotrienes (CysLTs) as well as CysLT receptor expression on human mast cells from healthy volunteers and patients with AD.

**Methods:**

Human mast cells were generated from CD34+ progenitor cells from peripheral blood. The cultured mast cells were stimulated with IL-4, IL-13, histamine and different histamine receptor selective ligands. Expression of enzymes in the biosynthesis of leukotrienes and expression of CysLT receptors were quantified by real-time PCR. The release of CysLTs was measured by ELISA.

**Results:**

Mast cells from AD patients showed higher expression of 5-Lipoxygenase (5-LO) and 5-Lipoxygenase activating protein (FLAP) compared to mast cells from healthy volunteers at baseline and in presence of histamine and TH2 cytokines. Expression of leukotriene C4 synthase (LTC4S), the biosynthesis of CysLTs, and mRNA expression of both CysLT receptors were induced by histamine and TH2 cytokines in mast cells from healthy volunteers and AD patients.

**Conclusion:**

We provide evidence that in an acute allergic situation histamine and TH2 cytokines may activate the biosynthesis of pro-allergic cysteinyl leukotrienes and up-regulation of CysLT receptor expression in human mast cells. This suggests a novel mechanism for sustaining mast cell activation through a possible autocrine signalling loop under these conditions.

**Supplementary Information:**

The online version contains supplementary material available at 10.1007/s00011-024-01974-6.

## Introduction

Atopic dermatitis (AD) is a chronic inflammatory skin disease with an increasing worldwide prevalence ranged from 2,1 to 4,9% in adulthood [1, 2]. It is defined by clinical criteria with a complex pathophysiology characterized by the involvement of genetic predisposition, skin barrier disruption, associated inflammation, environmental factors and immune dysregulation [3]. A characteristic symptom of AD is itching which is more closely correlated with patient-reported outcomes than with objective assessments by the physician and represents a significant burden for patients [4].

During acute inflammation the mRNA expressions of the TH2 cell associated cytokines IL-4, IL-5 and predominantly IL-13 are significantly up-regulated in skin lesions from AD patients when compared to the uninvolved skin [5–7].

Single-cell RNA and T-cell receptor sequencing on immune cells enriched from skin biopsies and matched blood samples of AD patients revealed that disease-specific T-cell clusters were mostly of a TH2/TH22 sub-population [8]. Beyond T-cells and other immune cells, mast cell numbers are also increased in the dermal infiltrate of the affected skin [9].

Mast cells, after IgE-mediated activation by IgE/FcεRI cross-linking, or after non-IgE-mediated activation, release a spectrum of mediators of allergic inflammation, such as histamine, prostaglandins, leukotrienes and cytokines [10, 11]. Treatments targeting IgE is approved for chronic spontaneous urticaria, but its efficacy in AD is inconclusive with varying results across studies [12, 13].

In contrast to histamine which is preformed in a one step reaction by decarboxylation of histidine predominantly in mast cells and basophils, cysteinyl leukotrienes (CysLTs) represent a class of immune-modulating eicosanoids which are rapidly synthesized de novo in a multi-step cascade in response to various stimuli mainly in basophils, eosinophils and mast cells and to a lower extend in macrophages and dendritic cells [14, 15]. Upon activation, free arachidonic acid (AA) is transferred to the complex of the 5-lipoxygenase (5-LO) associated with 5-lipoxygenase activating protein (FLAP) which is located at the perinuclear membrane [16, 17]. One of the formed products after converting AA by the 5-LO/FLAP complex is leukotriene A4 (LTA4) which is converted by leukotriene C4 synthase (LTC4S) to leukotriene C4 (LTC4). The additional CysLTs LTD4 and LTE4 are generated via sequential cleavage of amino acids from the glutathione moiety of LTC4. The functions of the CysLTs are mediated via two known G protein-coupled receptors: the CysLT type 1 receptor (CysLT1R) and the CysLT type 2 receptor (CysLT2R) [17–19].

Beyond basophils, which have the greatest capacity, eosinophils and, also mast cells synthesize and release CysLTs upon activation by allergens, cytokines, or other stimuli in allergic disorders [15]. CysLTs belong to the important inflammatory components in the pathophysiology of asthma bronchiale. They induce increased microvascular permeability leading to pulmonary edema and increased mucus secretion. Furthermore, they induce smooth muscle contraction and are recognized as the most potent known bronchoconstrictors [16]. In lesional skin of AD leukotrienes have been detected to be significantly increased [20]. Several studies consistently demonstrate that AD patients, in particular those with moderate to severe disease, have significantly elevated urinary LTE4 levels compared to healthy volunteers, indicating increased systemic production of CysLTs [21, 22].

Also histamine is present in elevated concentrations in AD skin lesions and regulates inflammatory and allergic responses. It binds to four different types of G protein-coupled receptors (H1R-H4R) [23]. In skin mast cells and in the human mast cell line 1 (HMC-1) the H1R, H2R and H4R are constitutively expressed at mRNA level. Quantitative analysis revealed that H2R and H4R mRNA expression exceeded that of H1R. However, H4R mRNA clearly shows the highest expression levels in skin mast cells [24, 25].

Previous research has been mainly focused on the process of histamine release from mast cells and the functional effects of histamine such as chemotaxis [26] or calcium mobilisation [27, 28] and chemokine or cytokine secretion [26]. Only few studies highlighted autocrine effects of histamine on mediator release of these cells so far [11, 29, 30].

For this reason, our study examined the influence of histamine and TH2 cytokines on the expression and regulation of the main components of the CysLT pathway, on the release of CysLTs and on the expression levels of their receptors on human mast cells derived from AD patients compared to cells from healthy volunteers.

## Materials and methods

### Study population

Peripheral venous blood was taken from healthy non-atopic volunteers (HVs) (n = 10, 6 male, 4 female, mean age 33 years, range 21–57 years) and AD patients (n = 10, 5 male, 5 female, mean age 39 years, range 30–66 years) recruited from the Department of Dermatology and Allergy, Hannover Medical School (Hannover, Germany). AD patients were defined following the diagnostic criteria established by Hanifin and Rajka [31]. The severity and activity of the disease was determined by the SCORAD index [32]. Patients showed moderate-to-severe disease activity (SCORAD mean 53, range 18–65). No patient in this study was under any systemic treatment for AD when the samples were taken. This study was approved by the local ethics committee of the Hannover Medical School (Vote 4253) and was conducted according to the Declaration of Helsinki Principles. All participants in the study including HVs gave their written informed consent.

### Isolation and cell culture of human mast cells

Peripheral blood samples served as source material for the isolation of human peripheral blood mononuclear cells (PBMCs). PBMCs were separated by density gradient centrifugation.

Mast cells were generated from CD34+  progenitor cells after positive immunomagnetic separation according to the manufacturer´s instructions (Miltenyi Biotec, Bergisch Gladbach, Germany). The isolated cells were collected and counted in trypan blue. Purified CD34+ cells were suspended at 0,5 × 10^6^ cells/ml and were plated into a 12-well plate in serum-free conditions using StemSpanTM medium (Stem Cell Technologies, Vancouver, Canada) supplemented with 10,000 U/ml penicillin and 10 mg/ml streptomycin (Biochrom AG, Berlin, Germany), human low-density lipoproteins (LDL) (5 ng/ml) (Stem Cell Technologies, Vancouver, Canada) and rhSCF (10 ng/ml) (R&D systems, Minneapolis, USA). Cytokines rhIL-3 (1 ng/ml) (R&D systems, Minneapolis, USA) and rhIL-6 (50 ng/ml) (ImmunoTools, Friesoythe, Germany) were added to the culture medium for 3 weeks after which IL-3 was omitted from the culture medium. The cells were cultured for 5 weeks in suspension for the entire period at 37 °C and 5% CO_2_ in a humidified atmosphere. The cytokine-supplemented medium was totally renewed weekly. Cell proliferation was estimated each week by counting the cells with a hemocytometer. Cell morphology was evaluated by toluidine blue staining. Cell purity was estimated by surface staining of the high-affinity IgE receptor (FcεRI) and CD117 (c-kit) using flow cytometric analysis.

### Histamine receptor ligands

The following histamine receptor ligands were used in this study: All histamine receptor ligands were used at a concentration of 10 µmol/l. Histamine (ALK-Abello, Madrid, Spain) as agonist for all histamine receptors; 2-pyridylethylamine as H1R agonist (Tocris Bioscience, Bristol, UK); amthamine as H2R agonist (Tocris Bioscience, Bristol, UK); ST-1006 as H4R agonist (Institute of Pharmaceutical and Medicinal Chemistry, Heinrich Heine University, 40225 Duesseldorf, Germany) [33]; JNJ7777120 as selective H4R antagonist (Sigma Aldrich, Deisenhofen, Germany). JNJ7777120 stands out among H4R antagonists due its selectivity, in comparison thioperamide a dual H3R/H4R antagonist, which has shown in experimental settings that it does not fully replicate the effects of JNJ7777120 in certain models [34, 35]. JNJ7777120 demonstrates a reliable blockade of immunological effects induced by the H4R agonist ST-1006 in human monocytes [36].

The 10 µmol/l concentration for H1R and H2R agonists which we used for stimulation human mast cells is commonly used and well established in pharmacological studies, and is supported by the literature [37]. In extensive previous studies we could show that the concentration of 10 µmol/l for histamine and the H1R/H2R and also for H4R agonists is a reliable way to study receptor activation related cellular responses in various immune cells [36, 38].

### Stimulation of human mast cells

Mast cells were stimulated with IL-4 (20 ng/ml), IL-13 (15 ng/ml) (R&D Systems, Wiesbaden, Germany), histamine, 2-pyridylethylamine (H1R agonist), amthamine (H2R agonist), ST-1006 (H4R agonist) for different time periods or left untreated. For blocking experiments mast cells were pre-incubated with the selective antagonists for the H4R (JNJ7777120) 30 min before stimulation. In preliminary experiments, we stimulated mast cells from HVs and AD patients with histamine for 24 h or left the cells non-stimulated. We activated the high-affinity IgE receptor (FcεRI) on histamine treated and untreated mast cells using antibodies directed against subunits of the FcεRI (Sigma Aldrich, Saint Louis, USA) for 30 min. 5-Lipoxygenase** (**5-LO), 5-LO activating protein (FLAP), leukotriene C4 synthase (LTC4S), cysteinyl leukotriene 1 receptor (CysLT1R) and cysteinyl leukotriene 2 receptor (CysLT2R) expressions were analysed by quantitative real-time PCR. Cysteinyl leukotrienes (CysLTs) release was analysed by ELISA.

### Real time quantitative PCR

Total RNA was isolated using the ReliaPrep RNA Miniprep Systems kit (Promega, Germany) according to the manufacturer’s instructions. The cDNA was synthesized by reverse transcription (Quantitect reverse transcription kit, Qiagen, Germany). Real-time quantitative PCR (qPCR) was performed using the LightCycler 480 (Roche Molecular Biochemicals, Mannheim, Germany) with Quantitect^®^ primer assays for 5-LO (ALOX5) (QT00015337), FLAP (ALOX5AP) (QT00077252), LTC4S (QT00000266), CysLT1R (QT00039368), CysLT2R (QT00216902) rps 20 (ribosomal protein S20) (QT00003290) and Primers from Tib Mol Biol (Berlin, Germany) for H2R (F: 5-TACCAgCTgTCCTgCAAgTg, R. 5´-CCCCAggTggATAgACAgAA) and H4R (F:5´-TgCTAggAAATgCTTTggTC, R:5´-gCgTgTgAgggATgTACAAA) using SYBR^®^ Green according to the manufacturer’s instructions (Qiagen, Hilden, Germany). The amount of the target mRNA relative to the amount of the reference gene mRNA, ribosomal protein S20 (rps 20), (target/reference ratio), in the same sample was calculated by the comparative Ct method using the Relative Quantification Software (Roche Molecular Biochemicals).

### Cysteinyl leukotrienes ELISA

Mast cells (2 × 10^5^ cells/well) were stimulated with histamine (10 µmol/l), IL-4 (20 ng/ml), IL-13 (15 ng/ml) for 48 h or left untreated. Cell free supernatants were collected for cysteinyl leukotrienes release which was estimated using the cysteinyl leukotrienes ELISA Kit (Cayman Chemicals, Michigan, USA) according to manufacturer´s instructions.

### Statistics

For statistical analyses, the software GraphPad Prism Version 8.0 was used (GraphPad software, San Diego, CA, USA). First, we performed methods to test the normal Gaussian distribution of the data. In cases where the normality tests passed, we performed the paired t-test and ANOVA Multiple Comparison test and the mean is shown in the graphs. In cases where the normality tests failed the Wilcoxon matched-pairs signed rank test or Friedman Dunn’s Multiple Comparison test were performed and the median is shown in the graphs. Comparisons between unpaired samples were performed by the Mann Whitney test. A *p* value < 0.05 was regarded as statistically significant (*p* < 0.05 was labelled with *p < 0.01 was labelled with **p < 0.001 was labelled with ***).

## Results

### Non-stimulated as well as histamine-, IL-4- and IL-13- stimulated mast cells from AD patients show higher mRNA expression levels of 5-lipoxygenase (5-LO) and 5-LO activating protein (FLAP) compared to cells from HVs

The eicosanoid metabolizing enzymes 5-LO and FLAP are acting jointly to convert AA to the unstable epoxide LTA4, which is the substrate for the leukotriene biosynthetic enzymes LTC4S and LTA4H [19]. We investigated a potential influence of histamine and TH2 cytokines on 5-LO and FLAP mRNA expression in CD34+  progenitor derived human mast cells from peripheral blood of HVs and AD patients. We stimulated the cells with histamine, IL-4, IL-13 or left them untreated.

Compared to untreated mast cells from HVs, we observed a trend towards up-regulation of 5-LO mRNA expression by histamine and IL-4. However, this trend was not detected in mast cells from AD patients. Both 5-LO and FLAP mRNA expressions were significantly down-regulated by IL-13 only in mast cells from HVs. In contrast, both 5-LO and FLAP mRNA expression levels showed almost equal amounts in non-stimulated and IL-13- stimulated mast cells from AD patients.

**Fig. 1:**
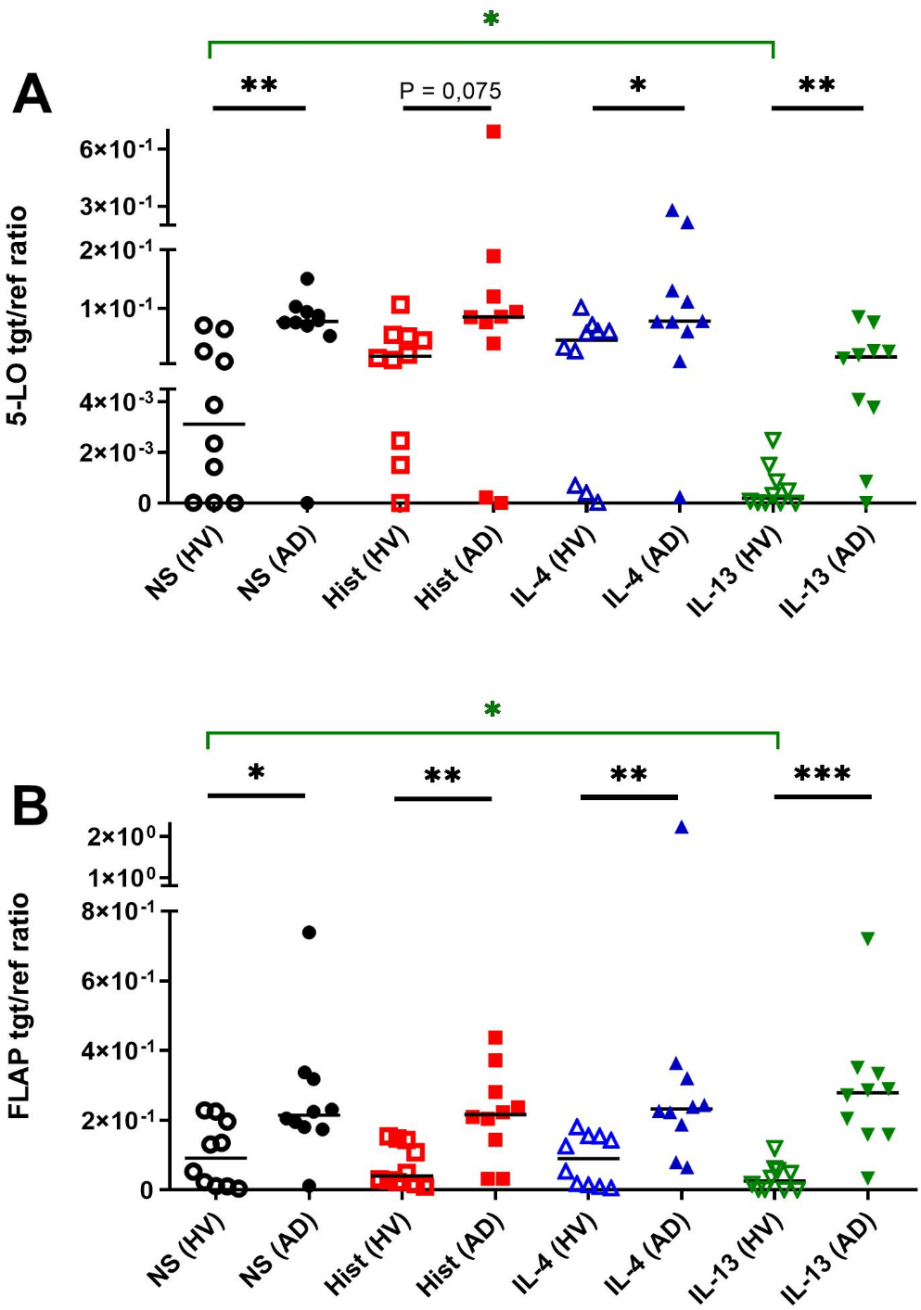
mRNA expression of 5-lipoxygenase (5-LO) and 5-LO activating protein (FLAP) in non-stimulated and stimulated mast cells from healthy volunteers (HVs) and atopic dermatitis (AD) patients. CD34 + progenitor derived mast cells from peripheral blood of HVs or AD patients were stimulated with histamine (Hist) (10 µmol/l), IL-4 (20 ng/ml), IL-13 (15 ng/ml) or left non-stimulated (NS) for 24 h. 5-LO (**A**) and FLAP (**B**) mRNA expression were analyzed by qPCR. Significant differences, as determined by Mann Whitney test (black bars) or by Wilcoxon matched-pairs signed rank test (green bars) are indicated as follows: *p <.05; **p <.01; ***p <.001. Data are shown as individual values with medians. A and B (n = 10); tgt/ref ratio = target/reference ratio

However, when comparing the mRNA expression of both targets between the two groups, we found the following differences: Mast cells from AD patients showed a significantly higher 5-LO mRNA expression in non-stimulated cells (24,9-fold) and in cells stimulated with IL-4 (1,70-fold) or IL-13 (85,1-fold) than in cells from HVs. Histamine stimulated cells from AD patients showed a tendency for higher 5-LO mRNA expression (4,8-fold) as compared to respective mast cells from HVs (Fig. 1A). Moreover, the expression of FLAP mRNA displayed a significantly higher expression in non-stimulated (2,3-fold), in histamine (5,3-fold), in IL-4 (2,5-fold) and in IL-13 (10,7-fold) stimulated mast cells from AD patients as compared to cells from HVs (Fig. 1B). The increased 5-LOX and FLAP mRNA levels in stimulated mast cells from AD patients compared to those from HVs are certainly attributable to higher baseline mRNA levels in untreated cells from AD patients. This conclusion is supported by the observation that mast cells from AD patients showed no up-regulation of 5-LOX and FLAP mRNA levels in response to stimulation with histamine, IL-4, or IL-13. The expression levels of 5-LOX, FLAP mRNA in non-stimulated conditions, and in stimulated conditions in mast cells from AD patients, were examined for correlation with the SCORAD, a widely accepted tool for evaluating disease severity of AD patients, using the Spearman correlation test. The Spearman correlation of 5-LOX and FLAP mRNA expressions with the SCORAD was weak and not statistically significant in all tested conditions.

### Histamine, IL-4 and IL-13 up-regulate leukotriene C4 synthase (LTC4S) mRNA expression in mast cells from HVs and AD patients

LTC4S is the final enzyme of the 5-lipoxygenase pathway responsible for the formation of LTC4. This homotrimeric transmembrane protein conjugates reduced glutathione to the unstable epoxide LTA4 to form LTC4, which is then transferred to the extracellular space [39].

**Fig. 2:**
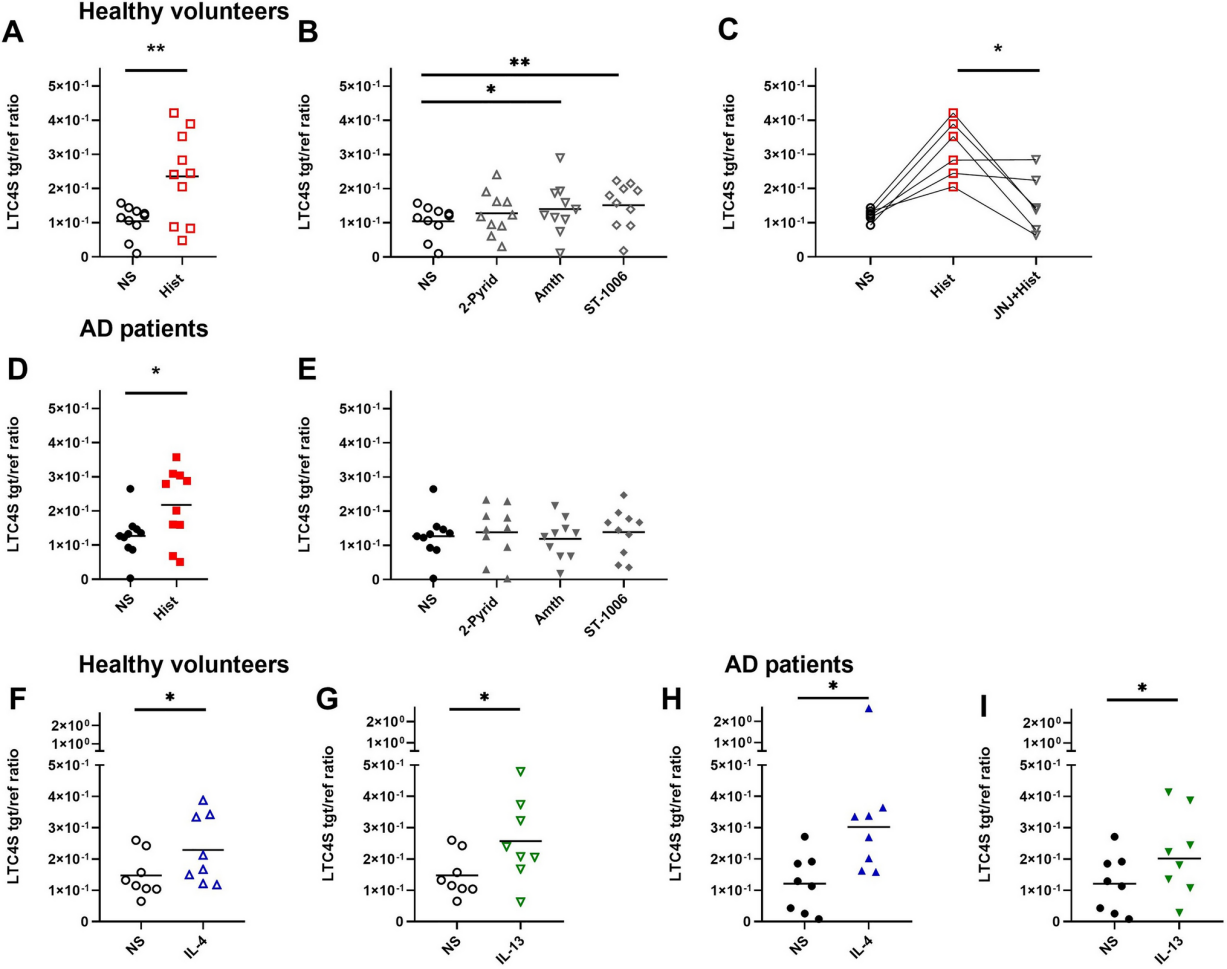
Histamine, IL-4 and IL-13 up-regulate leukotriene C4 synthase (LTC4S) mRNA expression in mast cells from healthy volunteers (HVs) and atopic dermatitis (AD) patients. CD34 + progenitor derived mast cells from peripheral blood of HVs (**A**–**C**) and AD patients (**D**–**E**) were stimulated with histamine (Hist), H1R agonist 2-pyridylethylamine (2-Pyrid), H2R agonist amthamine (Amth), H4R agonist (ST-1006) or left non-stimulated (NS) for 24 h. (**C**) The histamine induced up-regulation was blocked by pre-incubation for 30 min with the selective H4R antagonist JNJ7777120 (JNJ). Only experiments with a robust up-regulation of LTC4S mRNA expression by histamine were included in the blocking experiments, (all ligands 10 µmol/l). Mast cells from HVs (**F** and **G**) and AD patients (**H** and **I**) were activated with IL-4 (20 ng/ml), IL-13 (15 ng/ml) or left non-stimulated (NS) for 48 h. LTC4S mRNA expression was detected by qPCR. Significant differences, as determined by paired t-test in A, C, D, F, G, I, Wilcoxon matched-pairs signed rank test in H or by ANOVA multiple comparison test in B were indicated as follows: *p <.05; **p <.01; Data are shown as individual values with means or medians in H and I. A, B, D, E (n = 10), C (n = 6), F-I (n = 8), tgt/ref ratio = target/reference ratio

Through stepwise cleavage of glutamate and glycine residues by means of transpeptidases and dipeptidases LTC4 is converted to LTD4 and LTE4 [16]. Therefore, we investigated the mRNA expression of LTC4S in mast cells from HVs and AD patients. The cells were subsequently stimulated with histamine or with the H1R agonist (2-pyridylethylamine), H2R agonist (amthamine), H4R agonist (ST-1006) or left untreated as indicated in Fig. 2A–E.

Remarkably, we observed a moderate but significant up-regulation of LTC4S mRNA expression in response to histamine (2,2- fold). This effect seemed to be mainly mediated via the selective H4R agonist ST-1006 (1,4- fold) in mast cells from HVs (Fig. 2A and B). Accordingly, the histamine-mediated up regulation of LTC4S mRNA expression in cells from HVs was inhibited by pre-incubation with the H4R selective antagonist JNJ7777120 (JNJ) (Fig. 2C). Histamine also significantly potentiated the LTC4S mRNA expression in mast cells from AD patients (Fig. 2D). The TH2 cytokines IL-4 and IL-13, induced a significant up-regulation of LTC4S mRNA expression in both groups. Up-regulation of LTC4S mRNA expression by IL-4 was more pronounced in stimulated mast cells from AD patients compared to cells from HVs (Fig. 2F and H), whereas IL-13 induced an almost similar up-regulation of LTC4S mRNA expression in mast cells from both groups (Fig. 2G and I).

### Histamine, IL-4 and IL-13 induce the release of cysteinyl leukotrienes (CysLTs) in mast cells from HVs and AD patients

**Fig. 3:**
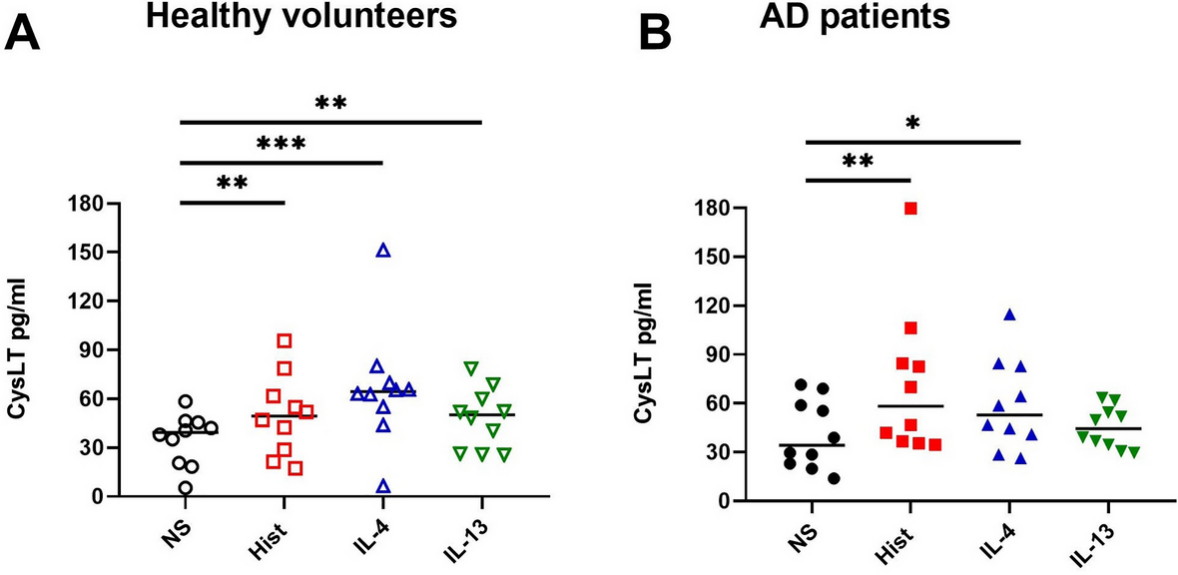
Histamine, IL-4 and IL-13 induce the release of cysteinyl leukotrienes (CysLTs) in mast cells from healthy volunteers (HVs) and atopic dermatitis (AD) patients. CD34 + progenitor derived mast cells from peripheral blood of HVs (**A**) or AD patients (**B**) were stimulated with histamine (Hist) (10 µmol/l), IL-4 (20 ng/ml), IL-13 (15 ng/ml) or left non-stimulated (NS) for 48 h. CysLT production was detected by ELISA. Significant differences, as determined by Friedman Dunn´s multiple comparison test are indicated as follows: *p <.05; **p <.01; ***p <.001; Data are shown as individual values with medians. A and B (n = 10)

Subsequently we analyzed whether the elevated mRNA levels of LTC4S would translate to increased biosynthesis of CysLTs. We stimulated CD34+ progenitor derived mast cells from peripheral blood of HVs (Fig. 3A) and AD patients (Fig. 3B) with histamine, IL-4 or IL-13. Release of CysLT secretion was investigated by ELISA. As expected from our findings on LTC4S mRNA expression we observed that the CysLT production was significantly up-regulated in samples stimulated with histamine and IL-4 in mast cells from both HVs and AD patients. IL-13 significantly up-regulated CysLT production only in cells from HVs. In preliminary experiments, we activated the high affinity IgE receptor (FcεRI) on histamine treated and untreated mast cells of two HVs and one AD patient using antibodies directed against subunits of the FcεRI for 30 min. We observed a strong induction of CysLT release due to the cross-linking of the FcεRI, which was further moderately enhanced by pre-stimulation with histamine, indicating a possible synergistic effect between FcεRI activation and histamine. Notably, in these preliminary experiments mast cells from the AD patient exhibited a higher release of CysLTs compared to those from the HVs, both in the non-stimulated samples and in the histamine- stimulated cells (Supplementary Fig. 1). It should be taken into account that these results are based on a very small number of samples.

### Histamine, IL-4 and IL-13 up-regulate cysteinyl leukotriene 1 receptor (CysLT1R) mRNA expression more strongly in mast cells from AD patients

Human tissue resident mast cells and cord blood derived mast cells express both types of CysLTRs, the CysLT1R and CysLT2R.

**Fig. 4:**
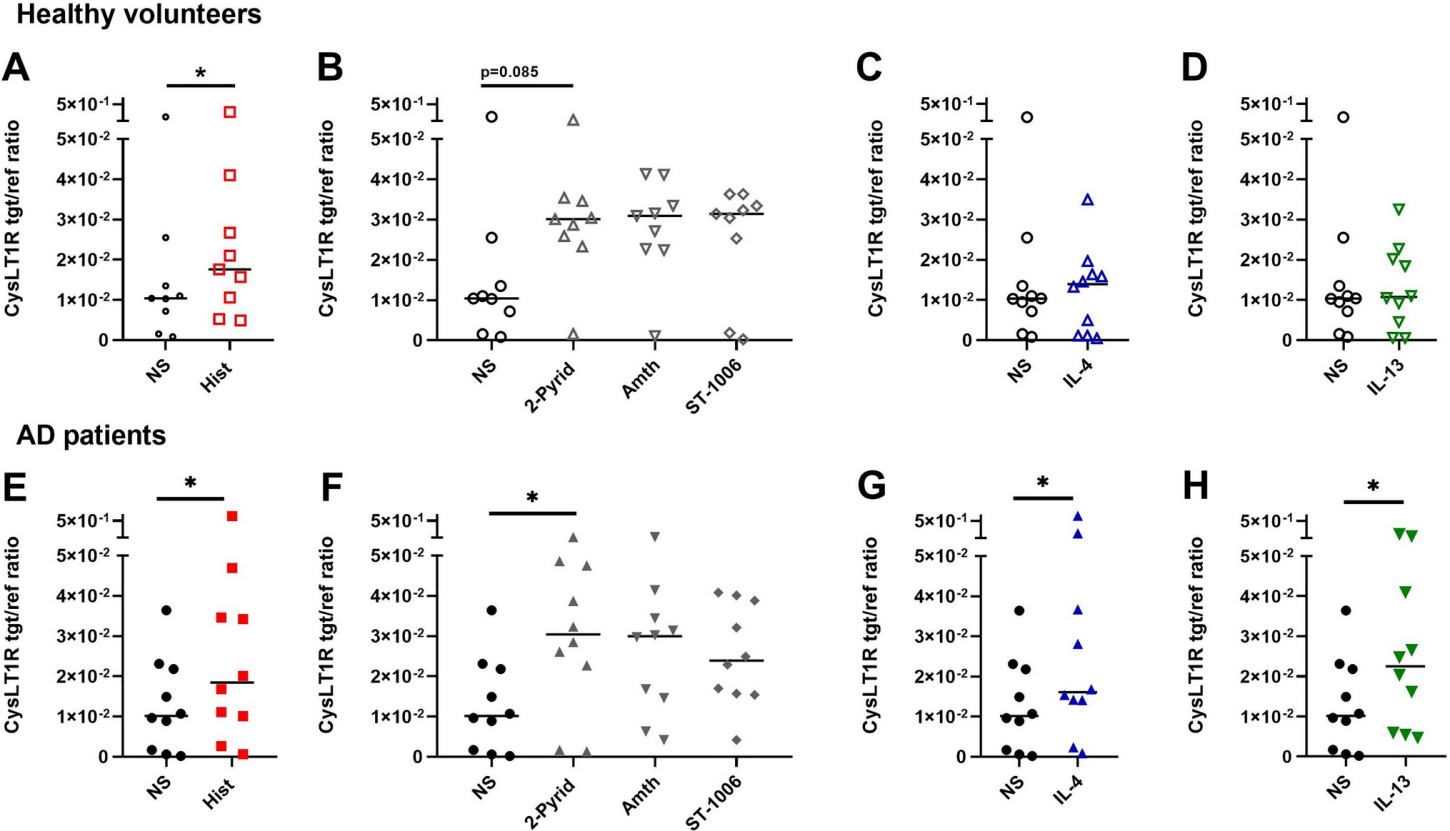
Histamine, IL-4 and IL-13 up-regulate cysteinyl leukotriene 1 receptor (CysLT1R) mRNA expression more strongly in mast cells from atopic dermatitis (AD) patients. CD34 + progenitor derived mast cells from peripheral blood of HVs (**A**–**D**) or AD patients (**E**–**H**) were stimulated with histamine (Hist), 2-pyridylethylamine (2-Pyrid), H2R agonist amthamine (Amth), H4R agonist (ST-1006) (all ligands 10 µmol/l) for 24 h, IL-4 (20 ng/ml), IL-13 (15 ng/ml) for 48 h or left non-stimulated (NS). CysLT1R mRNA expression was detected by qPCR. Significant differences, as determined by Wilcoxon matched-pairs signed rank test in A, E, G, H or by Friedman Dunn´s multiple comparison test B, F are indicated as follows: *p <.05. Data are shown as individual values with medians. A, B (n = 9) C-H (n = 10), tgt/ref ratio = target/reference ratio

We investigated whether histamine, selective agonists targeting the different histamine receptors or the TH2 cytokines IL-4 and IL-13 regulate CysLT1R and CysLT2R mRNA expression levels in mast cells. Therefore, we stimulated CD34+ progenitor derived mast cells from peripheral blood of HVs (Fig. 4A–D) or AD patients (Fig. 4E–H) or left the cells untreated. We could show that histamine significantly up-regulated the CysLT1R mRNA expression in mast cells from HVs and AD patients. The effect was mainly mediated via H1R in both groups. However, a significant up-regulation of CysLT1R mRNA expression by H1R stimulation was only observed in mast cells from AD patients. IL-4 and IL-13 up-regulated CysLT1R mRNA expression only in mast cells from AD patients but not in cells from HVs.

### Histamine up-regulates cysteinyl leukotriene 2 receptor (CysLT2R) mRNA expression in mast cells from HVs and AD patients. IL-4 and IL-13 up-regulate CysLT2R mRNA expression more strongly in mast cells from AD patients

First, we analysed the baseline CysLT2R mRNA expression in untreated mast cells from AD patients and HVs. We stimulated the mast cells of both groups with histamine, selective agonists targeting the histamine receptors and with TH2 cytokines. 

**Fig. 5:**
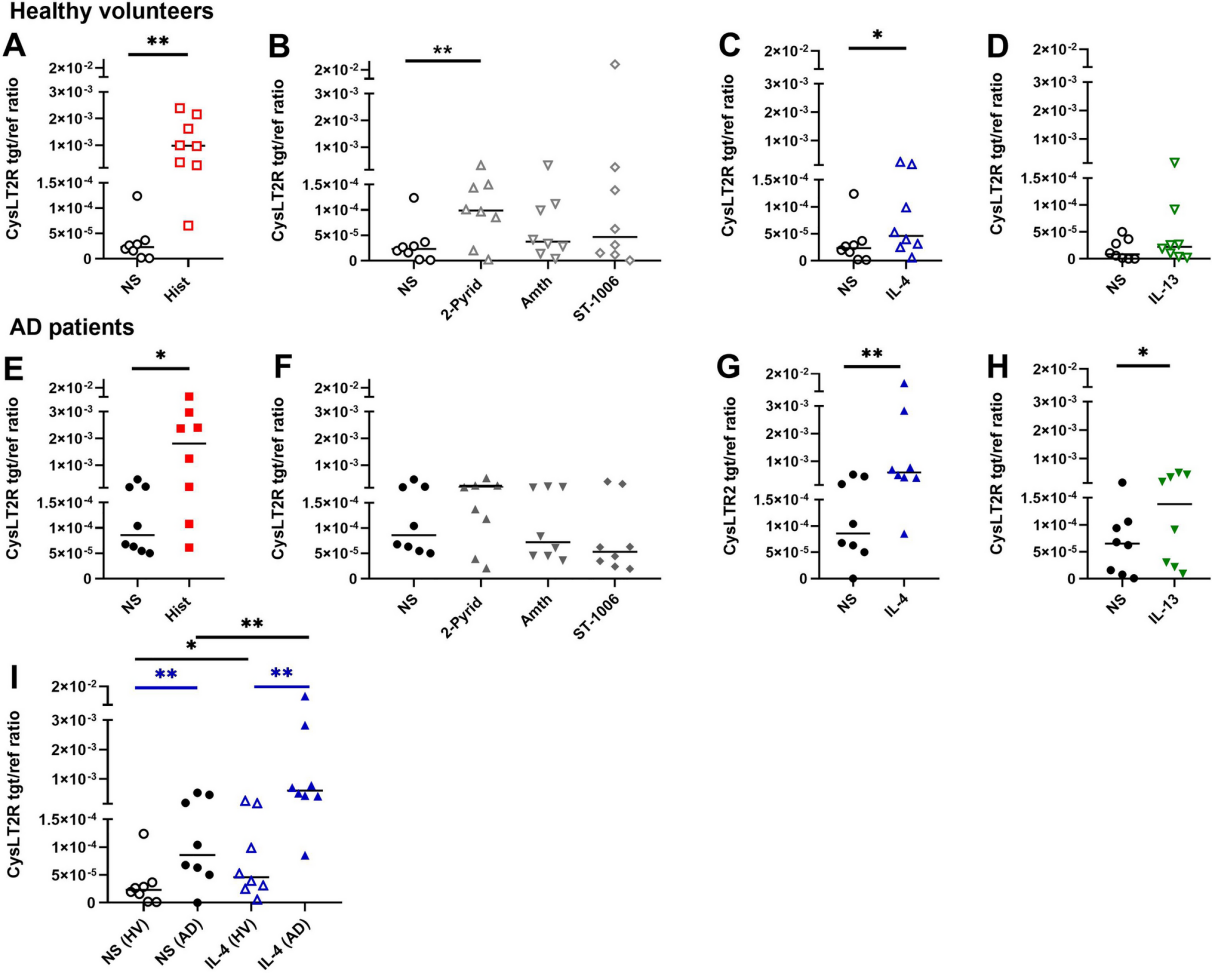
Histamine, IL-4 and IL-13 up-regulate cysteinyl leukotriene 2 receptor (CysLT2R) mRNA expression in mast cells from healthy volunteers (HVs) and in mast cells from atopic dermatitis (AD) patients. CD34 + progenitor derived mast cells from peripheral blood of HVs (**A**–**D**) or AD patients (**E**–**H**) were stimulated with histamine (Hist), 2-pyridylethylamine (2-Pyrid), H2R agonist amthamine (Amth), H4R agonist (ST-1006) (all ligands 10 µmol/l) for 24 h, IL-4 (20 ng/ml), IL-13 (15 ng/ml) for 48 h or left non-stimulated (NS). (**I**) shows the CysLT2R mRNA expression from (**C**) (HVs) and (**G**) (AD patients) with the significance levels between the two groups in direct comparison. CysLT2R mRNA expression was detected by qPCR. Significant differences, as determined by Wilcoxon matched-pairs signed rank test in A, C, E, G, H, I; by Friedman Dunn´s multiple comparison test in B or by Mann Whitney test in I (blue bars) are indicated as follows: *p <.05; **p <.01. Data are shown as individual values with medians. A–I (n = 8), tgt/ref ratio = target/reference ratio

Histamine, mainly via H1R agonist, significantly up-regulated CysLT2R mRNA expression in mast cells from HVs (Fig. 5A and B) and by trend regarding the stimulation of the H1R in mast cells from AD patients (Fig. 5E and F). IL-4 up-regulated CysLT2R mRNA expression in mast cells from AD patients to a higher degree than in cells from HVs (Fig. 5C and G). IL-13 significantly up-regulated CysLT2R mRNA expression in mast cells from AD patients (Fig. 5H) but not in cells from HVs (Fig. 5D). Remarkably, we observed that CysLT2R mRNA expression in non-stimulated (baseline expression) and in IL-4-stimulated mast cells show significantly higher levels in mast cells from AD patients as compared to cells from HVs (Fig. 5I).

The expression levels of CysLT2R mRNA in non-stimulated conditions, and in stimulated conditions in mast cells from AD patients, were examined for correlation with the SCORAD.

The Spearman correlation in the stimulated conditions was weak and not significant. For the baseline expression of CysLT2R mRNA we calculated a Spearman correlation coefficient of 0.585, indicating a moderate to strong positive correlation between AD severity and CysLT2R mRNA expression.

While not reaching statistical significance, this finding suggests a potentially meaningful relationship between CysLT2R expression in mast cells and the severity of atopic dermatitis as measured by SCORAD. This result may imply that as the severity of AD increases (higher SCORAD values), there tends to be a corresponding increase in CysLT2R expression in mast cells.

### Higher H2R and H4R mRNA expression in mast cells from AD patients than in cells from HVs

**Fig. 6:**
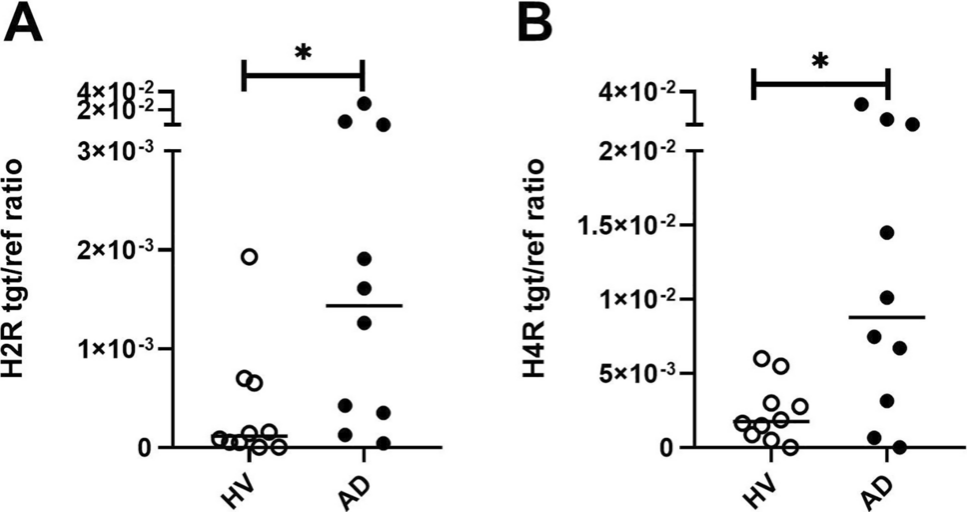
Histamine H2 receptor (H2R) and histamine H4 receptor (H4R) mRNA expression in mast cells from atopic dermatitis (AD) patients and from healthy volunteers (HVs). The basal mRNA expression levels of the H2R (**A**) and H4R (**B**) in CD34 + progenitor derived mast cells from peripheral blood of HVs or AD patients are shown. The HXR mRNA expression was detected by qPCR. Significant differences, as determined by Mann Whitney test are indicated as follows: *p <.05. Data are shown as individual values with medians. A and B (n = 10), tgt/ref ratio = target/reference ratio

We investigated the expression levels of histamine receptors H2R and H4R in human mast cells from HVs and AD patients. We could demonstrate that mast cells from AD patients showed a significantly higher baseline mRNA expression of H2R (12,5-fold) and H4R (5,0-fold) when compared to mast cells from HVs (Fig. 6A and B).

## Discussion

**Fig. 7:**
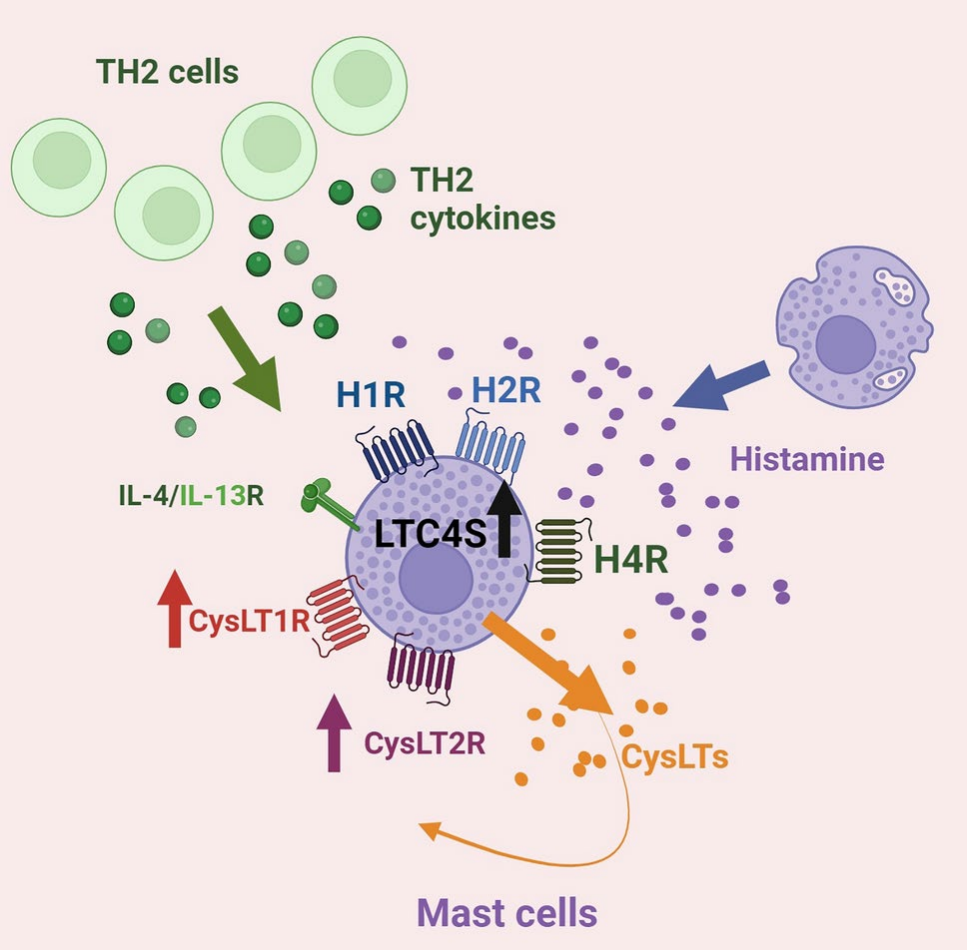
In skin lesions of atopic dermatitis (AD) mast cells beyond other immune cells are present in increasing numbers. Among the various receptors involved in atopic dermatitis (AD) pathogenesis mast cells express the histamine receptors histamine-1-receptor (H1R), H2R, and H4R, the cysteinyl leukotriene receptors CysLT1R and CysLT2R, as well as the receptors for IL-4 and IL-13. Histamine, mainly released from activated mast cells, or TH2 cytokines, which are abundant in acute AD skin, up-regulate leukotriene C4 synthase (LTC4S) mRNA expression, induce the release of cysteinyl leukotrienes (CysLTs), and differentially up-regulate the mRNA expression of CysLT receptors in mast cells providing a novel mechanism for sustaining their activation through a possible autocrine signalling loop (schematic overview based on the results shown here) (created by BioRender.com)

Mast cells play a decisive role in the pathophysiology of AD. In the skin these bone marrow-derived cells are located adjacent to blood and lymphatic vessels, glandular structures or hair follicles. Furthermore, the connection of some mast cells with nerve endings was observed. In chronic lesional skin of AD patients mast cell numbers are increased and the cells are activated by the inflammatory microenvironment [24, 25]. Vice versa through production of cytokines, chemokines or growth factors the cells regulate recruitment and functions of other immune cells and contribute to the inflammatory response. Mast cells in AD express several key receptors on their cell surface that mediate their activation and function in the disease. The FcεR1 triggers IgE mediated mast cell degranulation, toll like receptors (TLRs) recognize bacterial components, the Mas-related-G-protein-coupled receptor X2 (MRGPRX2) is involved in IgE independent activation by neuropeptides, IL-4 and IL-13 receptors type I and II respond to these cytokines [14]. These receptors enable mast cells in the atopic microenvironment to respond to different stimuli with the release of inflammatory mediators, in particular histamine and CysLTs [11, 40]. Although both TH2 cells and group 2 innate lymphoid cells are considered as the primary cellular sources of IL-4 and IL-13 in atopic dermatitis skin, mast cells also contribute to the elevated levels of these TH2 cytokines [5, 41] (Fig. 7).

In this study we investigated the effects of histamine and TH2 cytokines on key components of the biosynthesis of CysLTs in human mast cells. The initial step of the biosynthesis of CysLTs from arachidonic acid is carried out by the enzyme 5-LO in interaction with the activation protein FLAP. We did not detect any significant up-regulation of these proteins by histamine or IL-4, IL-13 in mast cells of both groups. Our findings are in accordance with previous publications who showed that priming of SCF treated human mast cells with IL-4 for 5 days leads to no apparent changes neither in 5-LO nor in FLAP expression [42].

The knowledge of the regulation of 5-LO and FLAP remains fragmentary, a selective up-regulation of 5-LO but not of FLAP by the glucocorticoid dexamethasone in human mast cells was observed [43]. In mouse bone marrow-derived mast cells IL-3 concomitantly increased the expression of 5-LO and FLAP [44].

However, we detected for the first time a significant higher 5-LO and FLAP mRNA expression in mast cells from AD patients when compared to respective cells from HVs. We suppose that priming of CD34+ progenitor cells by mediators which are elevated in the blood of AD patients or epigenetic effects on progenitor cells provide the basis for the increased levels of 5-LO and FLAP expression in the patient group which is not lost during the in-vitro differentiation to mast cells. Allakhverdi et al. stated that blood CD34+ cells expressing the receptors for TSLP and IL-33, in addition to being progenitors, may act as pro-inflammatory effector cells [45]. CD34+ cells respond to TSLP and IL-33 by releasing TH2 cytokines and chemokines thereby these progenitor cells directly contribute to the allergic inflammation [45].

A possible role of hematopoietic progenitor cells in establishing cellular inflammation in allergic diseases such as rhinitis, asthma or eczema was described in a pilot study showing increased CD34+ cell traffic in those patients as compared to non-allergic controls [46].

Zileuton is the only selective 5-lipoxygenase inhibitor approved as a treatment for asthma also used as a selective tool to evaluate the role of 5-LO and CysLTs. Its function has been studied in several mouse models and shown to have effects on inflammation and disease processes through inhibition of the 5-LO pathway [47]. In a pilot study the role of zileuton was examined in six adult AD patients over 6 weeks. It was observed that the disease severity scores including pruritus showed a trend toward improvement following zileuton treatment [48].

Converting AA by 5-LO and FLAP leads to the formation of the short lived intermediate LTA4 as precursor for leukotriene synthesis. LTC4S plays a central role in the generation of CysLTs [49]. We detected an up-regulation of LTC4S mRNA expression and CysLT liberation upon histamine or TH2 cytokine stimulation in mast cells from AD patients and from HVs. The constitutive expression levels were approximately the same in both groups.

Our findings indicate no difference in the expression of LTC4S between the two groups: AD patients and HVs. Therefore, we conclude, in agreement with the study by Di Gennaro et al., [50] that LTC4S plays a critical role as a potential rate-limiting factor in the final phase of LTC4 synthesis and subsequently for other CysLTs. Since both groups exhibit equivalent levels of LTC4S mRNA, we anticipate that their leukotriene release profiles would be similar [50]. Hsieh et al. detected a dramatic up-regulation of LTC4S transcripts and protein by IL-4. In contrast to our observations IL-13 failed to augment LTC4S expression in their study [42]. Of note Hsieh et al. used human mast cells pre-treated with SCF and stimulated the cells for 6 h with TH2 cytokines [42] instead for 48 h in our protocol. We assume that the different time periods in both studies play a role for the regulation of the LTC4S at transcriptional levels in response to IL-4 or IL-13.

Induction of LTC4S protein expression by IL-4 was also detected in wild-type mouse bone marrow-derived mast cells within 72 h. Further it was shown that IL-4 which induces LTC4S expression in human mast cells requires CysLTs to promote mast cell proliferation leading to the assumption that autocrine actions of endogenous CysLTs are involved in this effect [51].

Further CysLTs have been shown to enhance release of various cytokines, including IL-8, TNF, and IL-1β contributing to the amplification of inflammatory responses [52, 53] or to down-regulate the expression of toll-like receptors in human mast cells [54]. These data show that CysLTs produced by mast cells themselves, may lead to autocrine signaling that further influences their activation and proliferation. This self-regulatory mechanism allows mast cells to amplify their response during allergic reactions or inflammatory processes. This modulation by CysLTs can affect how mast cells respond to various stimuli, potentially altering their activation state and the subsequent inflammatory response.

Here we could show that histamine, IL-4 or IL-13 without FceRI engagement activate the release of elevated levels of CysLTs from human mast cells obtained from HVs and AD patients. We wanted to capture the total production of CysLTs over a 48 h period rather than peak levels since newly synthesized CysLTs can continue to be produced and released for several hours after the initial stimulation. We acknowledge that the 48 h incubation period likely represents an underestimate of total production, which could be a limitation to consider when interpreting these results.

Mast cell derived CysLTs could play a role in acute and chronic itch and hyperkeratosis. It has been demonstrated in a MC908 mouse model that coupling of LTC4 with its receptor CysLT2R, which is expressed in mice and in humans in a population of peripheral sensory neurons, represents a potent itch inducer [55, 56]. In ovalbumin sensitized skin of mice CysLTs promote hyperkeratosis and fibrosis of allergic skin inflammation [57].

We detected that mast cells from AD patients express higher mRNA levels of H2R and H4R when compared to cells from HVs. The differential expression levels of histamine receptors on mast cells have been well documented in the literature and are thought to play a crucial role in their varied functional effects [26].

It is also important to highlight the distinct affinities that histamine exhibits for these receptors. While H2R have a low affinity in the micromolar range, H4R are high-affinity histamine receptors with affinities in the nanomolar range [58].

In general, the H2R demonstrates predominantly anti-inflammatory effects: In T-cells, H2R activation can suppress certain T-cell responses and may favor TH2 polarization and production of IL-10 [59]. H2R activation inhibits mast cell [60] and basophil [61] degranulation and leukotriene and histamine release, contributing to the stabilization of these cells. Chemotaxis and calcium influx in mast cells could only be blocked by selective H4R antagonists, not by H2R antagonists [26]. In contrast functional activation of H4R on mast cells leads to the production of inflammatory mediators. However, in basophils, the H4R also mediates a reduced release of leukotrienes [61]. In agreement with our work, Jemima et al. [25] demonstrated that both histamine and 4-methylhistamine (H4R/ H2R agonist) induced the release of CysLTs and LTB4 in cord blood-derived CD34 + human mast cells. Furthermore, the release of various cytokines and chemokines associated with inflammatory diseases via H4R from human mast was also described in their work [25]. The distinct roles of H2R and H4R on mast cells highlight the complexity of histamine signaling in inflammatory conditions like atopic dermatitis.

However, after stimulation of both receptors H2R and H4R an up-regulation of LTC4S was only observed in cells from HVs.

The fact that histamine up-regulated the leukotriene receptors CysLT1R and CysLT2R in AD patients underscores the influence of both mediators histamine and leukotrienes and their receptors as effector targets on mast cells in the allergic milieu. Histamine up-regulates CysLT1R and CysLT2R mRNA expression mediated by trend via the H1R. We suggest that the difficulty to attribute the up-regulation of CysLT1R and CysLT2R mRNA expression to specific histamine receptor subtypes arises from their overlapping functions and the complexity of histamine signaling. Each receptor subtype can influence similar pathways, potentially leading to different or similar outcomes [58].

Interestingly, studies from Pynaert et al. implicate that CysLTs vice versa act as possible inducers of increased histamine responses by up-regulating the H1R expression [62].

Taken together, we provide evidence that histamine and TH2 cytokines may activate the expression of enzymes in the biosynthesis of CysLTs resulting in enhanced release of CysLTs and up-regulation of CysLT receptor expression suggesting a novel mechanism for sustaining mast cell activation. The interplay of the histaminergic system and the leukotriene system, with histamine and leukotrienes as key mediators in the atopic environment, offers promising therapeutic targets. This interaction suggests potential for developing treatments that may inhibit mediator production in both systems.

## Supplementary Information

Below is the link to the electronic supplementary material.

Supplementary file1 (DOCX 38 KB)

## Data Availability

A data availability statement for this journal is provided by the authors.
