# Supplementary material for: Histamine and TH2 cytokines regulate the biosynthesis of cysteinyl-leukotrienes and expression of their receptors in human mast cells
Source: Inflamm Res. 2025 Jan 31;74(1):32. doi: 10.1007/s00011-024-01974-6 (PMC11785601; doi:10.1007/s00011-024-01974-6)
Supplement: Supplementary file 1 — Supplementary file1 (DOCX 38 KB) [file 11_2024_1974_MOESM1_ESM.docx]

**Supplementary Material**

**
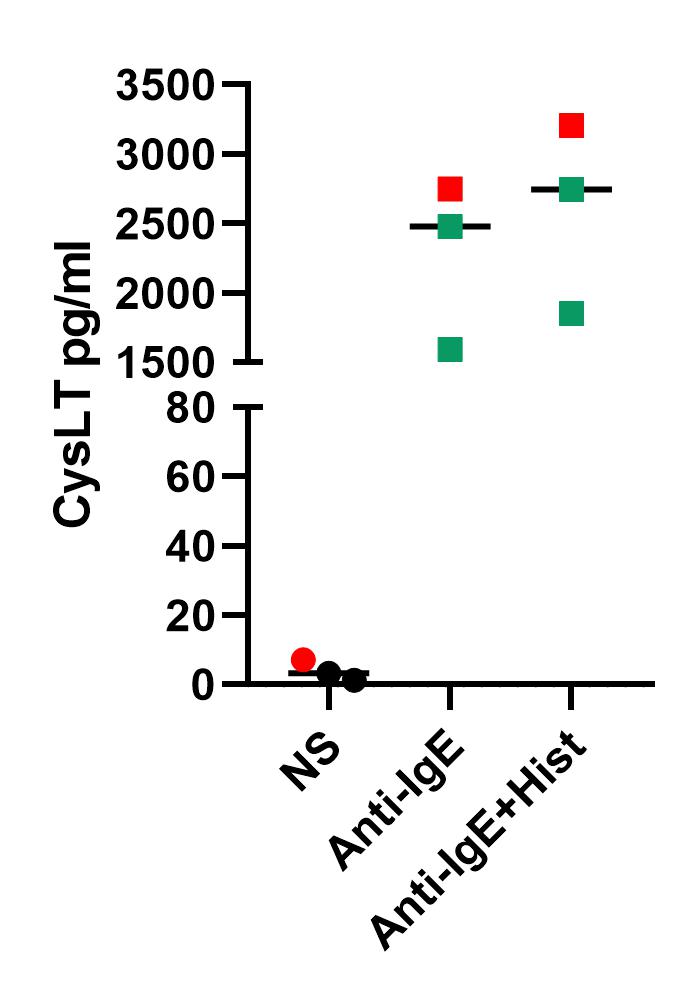
**

**Supplementary Figure 1: Activation of the high affinity IgE receptor (FcεRI) highly increased CysLT release, which was slightly enhanced in mast cells pre-treated with histamine from healthy volunteers (HVs) and atopic dermatitis (AD) patients.**

Human CD34+ progenitor derived mast cells from peripheral blood of HVs or AD patients were pre-incubated with histamine (Hist) (10 µmol/l) for 24 h or left non-stimulated (NS). The high affinity IgE receptor (FcεRI) was activated with antibodies (Anti-IgE) (100 ng/ml) directed against the ε chain of the FcεRI for 30 min. CysLT production was detected by ELISA. Data are shown as individual values with medians. (n = 3, 2 HVs, 1 AD patient marked in red).
